# Functionalization of Artificial Freestanding Composite Nanomembranes

**DOI:** 10.3390/ma3010165

**Published:** 2010-01-07

**Authors:** Zoran Jakšić, Jovan Matovic

**Affiliations:** 1 Center of Microelectronic Technologies and Single Crystals (CMTM), Institute of Chemistry, Technology and Metallurgy (IHTM), University of Belgrade, Njegoševa 12, 11000 Belgrade, Serbia; 2 Institute for Sensor and Actuator Systems (ISAS), Faculty of Electrical Engineering & Information Technology, Vienna University of Technology, Gusshausstrasse 27-29/366-MST, A-1040 Vienna, Austria; E-Mail: Jovan.Matovic@tuwien.ac.at (J.M.)

**Keywords:** metal-matrix nano/nano-composites, nanomembranes, nanosystem technologies, gated ion channels, nanoparticle fillers, synthesis, processing, structural and functional properties

## Abstract

Artificial nanomembranes may be defined as synthetic freestanding structures with a thickness below 100 nm and a very large aspect ratio, of at least a few orders of magnitude. Being quasi-2D, they exhibit a host of unusual properties useful for various applications in energy harvesting, sensing, optics, plasmonics, biomedicine, *etc.* We review the main approaches to nanomembrane functionalization through nanocompositing, which ensures performance far superior to that of simple nanomembranes. These approaches include lamination (stacking of nanometer-thin strata of different materials), introduction of nanoparticle fillers into the nanomembrane scaffold, nanomembrane surface sculpting and modification through patterning (including formation of nanohole arrays and introduction of ion channels similar in function to those in biological nanomembranes). We also present some of our original results related to functionalization of metal matrix composite nanomembranes.

## 1. Introduction

Recently a special class of artificial materials has revealed itself as a novel and attractive topic in the wide field of nanotechnologies: the nanomembranes, whose structures are at the same time ultrathin (on the order of nanometers), with large lateral dimensions (of the order of millimeters, even centimeters) and sufficiently robust to stand freely, without additional support or substrate [1,2,3]. Although their natural counterparts have been present since the dawn of time, a prototype example being the biological nanomembrane making up the protective cover of living cells [4], and artificial ones (for instance, Langmuir-Blodget films) have been well known for at least several decades [5], it was only recently that they attracted considerable attention among the scientific community.

The trigger to a sudden expansion of the nanomembrane field was the possibility of using nanotechnologies to accurately control and modify nanomembrane properties at the nano-level. In other words, novel technologies provided the possibility to functionalize the already known but poorly applicable structures, to design new ones and to tailor both to specific needs. This exposed artificial (synthetic) nanomembranes as attractive novel nano-building blocks. Without functionalization, typically done through nanocompositing, one gets only what blind chance gives, and which may be useful, or more often is not. As in many other situations, a smart design opens many new degrees of freedom.

The most widespread natural nanomembranes are surely the lipid bilayers which make up the protective outer cover of the living cells, from the simplest organisms to the most complex ones, including humans [5] and life as we know it would not be possible without biological nanomembranes, yet in spite of their ubiquitous nature and primordial origin, living nanomembranes are limited to a relatively narrow range of protein-based biological materials.

In contrast to the lipid bilayer membranes, the man-made structures (artificial—synthetic nanomembranes) theoretically can be fabricated from a much wider range of materials, both inorganic and organic. They do include as a subgroup the materials found in living organisms, however they are by no means limited only to them and many materials never found in living structures and actually sometimes even incompatible with them may be used. For instance, one could readily imagine structures containing e.g., nanoparticles of isotopes emitting ionizing radiation, even at lethal doses. In addition to that, the artificial nanomembrane material toolbox does not even have to be limited to structures and properties found in Nature, an example to this being the electromagnetic metamaterials with negative refractive index [6]. However, in spite of the much wider material toolbox and high potential, the applicability of the currently produced synthetic nanomembranes is very poor. The absence of complex functionalities in these structures, even at a rudimentary level, is striking. The versatility and complexity of biological structures surpasses by far anything man has ever produced.

The key is the immense multifunctionality added to the biological nanomembranes. There are numerous structures, some of them exceedingly complex, built into the cell walls. A lipid bilayer itself, the basic constituent of a cell membrane, is readily produced artificially in its simple form (e.g., [7]). However, the ion channels built into the cell walls are those that actually enable the exchange of matter between the living cell and its exterior [8]. If they are disturbed or disabled, the cell ceases its function. In this case, the functionalization of the nanomembrane is the difference between a living thing and an inanimate object.

Functionalization in synthetic nanomembranes likewise represents an essential step towards fundamental extension of their applicability. Basically, this includes imparting upon the nanomembrane additional desirable mechanical, electronic, chemical, biological, optical, magnetic, *etc.* properties or any of their combinations. The way to do that is obviously nanocompositing with different kinds of functional building blocks.

One of the problems is that nanomembranes are freestanding (possibly free-floating) structures with a thickness very close to the fundamental limits of the solid matter; they may be a few tens of atomic/molecular layers thick, down to freestanding monatomic layers. For this reason many methods customarily used in MEMS and nanoscience obviously cannot be applied since they are simply too intrusive. Nanomembranes can hardly be patterned utilizing micrometer-thick photoresist coating, and many methods making use of directed energy or particles would quickly destroy them. If fact, caution should be taken even whilst imaging the samples by SEM, since for instance the use of electron beam energies in excess of 5kV results in damages to the metal-composite nanomembrane surfaces. Thus, methods for functionalization of nanomembranes have to be very carefully chosen.

In this article we present an overview of the main methods for nanomembrane multi-functionali­zation from the point of view of fabrication of various nanocomposites. We offer a possible classification of the currently used approaches. We provide a review of the state of the art works, without any pretense to a generalization. After a short consideration of the simple (non-functionalized) nanomembranes, we present various methods to impart novel functions. These include layering (stratification, lamination), surface activation, patterning, pore forming, introduction of nanofillers and 3D sculpting (surface modulation).

## 2. Artificial (Synthetic) Nanomembranes Overview

Nanomembranes generally form a subgroup among a larger group of membranes. From the point of view of the theory of elasticity, membranes balance the external pressure exclusively by in-plane membrane stresses, *i.e.,* no bending rigidity components of the stress are present [9]. A possible and somewhat arbitrary definition of a membrane is a freestanding or free-floating self-supported structure whose width-to-thickness and length-to-thickness aspect ratios both exceed 100. A nanomembrane may be then defined as a membrane with a thickness below 100 nm, the thinnest structures being on the monomolecular or monatomic level. It can be seen that in the given definition no limitations are posed regarding the nanomembrane composition. In literature, such structures are variably termed ultrathin freely suspended films or simply ultrathin structures, unbacked films, freestanding films, freestanding membranes, self-supported films, suspended nanofilms, atomic membranes, monolayer membranes, *etc.* If the dimensionality is obvious from the context, they are often simply denoted as membranes.

We should stress a terminological point here. In the literature, one often encounters the term nanomembrane representing a porous membrane with a thickness that may be of the order of micrometers, even hundreds of micrometers, but which is mesoporous or microporous [10]. Thus, the existence and dimensions of pores serve to identify the membrane instead of its dimensions and thus sometimes the word 'nanomembrane' denotes a structure almost a millimeter thick and with a surface which may be on the order of square decimeters.

Throughout this text we use the term 'nanomembrane' exclusively to denote a freestanding film with a thickness below 100 nm. The bottom physical limit to the freestanding nanomembrane thickness is obviously a molecular or atomic monolayer, *i.e.,* about 0.3 nm.

Nanomembranes are simultaneously nano-objects (because of their thickness and their low-dimensional physics and chemistry) and macroscopic or at least microscopic objects (because of their large lateral dimensions). In fact, typical nanomembranes, with lateral dimensions of the order of millimeters, or even centimeters, are among the very rare nanotechnological objects which may be manipulated without special equipment and observed with a naked eye.

Nanomembranes are the nearest objects to ideal 2D structures. Monatomic freestanding, self-supported layers have already been demonstrated [11], and freestanding, self-supported nanomembranes 15–30 atomic or molecular layers thick and with mm^2^, even cm^2^ areas are readily produced [12,13,14].

Some physical properties of freestanding, self-supported nanomembrane are fundamentally different from those of the thick or bulk objects made of the same material. This arises mostly due to the dimensional confinement. Quantum effects are marked in nanomembranes. Their electrical and thermal conductivity will differ from those of bulk structures of the same material, sometimes by several orders of magnitude. Their electromagnetic and optical behavior will be modified because of the mutual influence of the two very close surfaces ­– an example being the appearance of long-range surface plasmons on metal nanomembranes [15]. The mechanical properties of ultrathin freestanding membranes are vastly different compared to thicker membranes. For instance, by their very nature nanomembranes have to be extremely compliant. They can be folded and wrapped to assume almost any shape from the micrometer level up and unfolded without consequences, furnishing for instance stretchable and bendable electronics made in ultrathin silicon [3].

As mentioned, nanomembranes are easily damaged by excess heat due to their minute thickness. Somewhat paradoxically, nanomembranes with smaller surface areas are easier to damage mechanically than their larger counterparts, which is a result of the mentioned ultra-compliancy and larger allowed absolute stretching at larger dimensions.

Simple (non-functionalized) nanomembranes may be divided into two large groups, the inorganic and the organic ones. Inorganic nanomembranes can be further separated into several main classes depending on their composition. The simplest one includes pure element nanomembranes and consists of three separate subclasses: pure metal structures, carbon-based nanomembranes – diamond and diamondoid (diamond-like) – and elemental semiconductor membranes, the key position being held by silicon, the main material for microfabrication/microelectromechanical systems (MEMS). Semiconductor combinations are also used (e.g., lattice-mismatched strained Ge on Si). Another large class includes simple inorganic compounds like oxides, nitrides, carbides and similar materials, most of which are mechanically robust, and many of which are semiconducting (e.g., gallium nitride, silicon carbide, *etc.*). Among the oxides, silicon dioxide occupies a central position in spite of its poorer mechanical characteristics, again because of its dominance in MEMS. The third important class includes glass and ceramic nanomembranes.

Probably the simplest nanomembranes are those consisting of pure metals. Some of the used materials include chromium, nickel, aluminum, platinum, palladium, silver, gold and similar. One also encounters titanium, tungsten, lead, tin, and copper. A common trait of these materials is that they are structural metals with better or worse mechanical characteristics. Most of them are used in microelectronics and in MEMS, but some of them are known for their use in catalysis. Their chemical inertness is usually rather good. Typically, the ultrathin pure metal membranes are fabricated utilizing the conventional microsystem technologies, *i.e.,* they used the well-known technique of sacrificial supporting structure [16]. Pure metal nanomembranes with surface areas up to about 10 mm^2^ have been obtained with aspect ratios up to 430,000:1 [16].

Carbon, diamond, and diamondoid (diamond-like) nanomembranes are another important group. They exhibit properties like chemical stability, wear resistance and optical transparency [17]. The hydrogenated forms of diamondoid films, a-C:H, have exhibited the lowest ever recorded friction coefficients, 0.001, as well as ultra-low wear rates of 10^−11^ to 10^−10^ mm^3^ N^−1^ m^–1^ [18]. The freestanding diamond membranes are readily combined with polymer, metal and other composite nanomembrane materials.

Oxide, nitride, carbide nanomembranes include various materials, of which silicon dioxide is most often met, other materials being TiO_2_, Ta_2_O_3_, MgF_2_, Y_2_O_3_, La_2_O_3_, ZrO_2_, *etc.* [19]. Silicon nitride is also frequently used. Most of these materials are mechanically robust. Typically, they are either dielectrics or wide bandgap semiconductors. Glasses and ceramics are also sometimes used as materials for experimental nanomembranes. They are applied as porous materials, thus enabling their application as nanosieves. Among the examples is sodium borosilicate glass, which was used to fabricate porous membranes with a thickness of about 100 nm.

Organic nanomembranes include various carbon-based macromolecules. There are an almost infinite number of macromolecular formulations for nanomembranes, which is naturally followed by a wide spectrum of useful chemical and physical properties. Typically, macromolecular nanomembranes are sensitive to humidity, temperature and solvents, have a low Young’s modulus and creep under permanent stress. Historically, collodion (pyroxylin) was the first macromolecular nanomembrane [20]. Many organic nanomembranes are produced by Langmuir-Blodgett and Layer-by-Layer techniques. Since these approaches allow fabrication of laminar structures of different materials, we will handle them in more detail later in the text, when dealing with nanomembrane multi-functionalization through layering. Among the organic nanomembranes, an important place belongs to phospholipid bilayers, the basic constituents of the living cell walls, which are also artificially produced by self-assembly techniques [7].

There are many available top-down and bottom-up methods for thin film fabrication (e.g., [21]). Usually some kind of support or substrate is utilized, giving mechanical strength and protection against overheating to the membrane and defining its shape. After the deposition process, the nanomembrane is freed from the support. Two generic approaches are observed among the membrane release procedures. One is to utilize some kind of sacrificial support on which the membrane material is deposited and to destroy that support at the end of the fabrication procedure. This support may be for instance a semiconductor wafer (silicon is probably the most often used, due to this ubiquitous presence in micro/nanofabrication), oxide layer (e.g., silicon dioxide), polymer, *etc.* It may be etched by a convenient etchant (for instance, if silicon is used, one may apply anisotropic etchants like KOH, EDP or TMAH, or isotropic etchants like HF/nitric/acetic acid (HNA) or dissolved (various organic solvents if polymers are used). A variation is the use of a permanent support over which an additional sacrificial layer is deposited. The second generic procedure is to leave the substrate intact, but to peel off the nanomembrane. A special case of this procedure is the fabrication of membrane on a liquid surface, where the membrane is simply lifted from the liquid at the end. The steps of freeing the membrane and releasing it from the solution often pose significant technological challenges.

**Figure 1:**
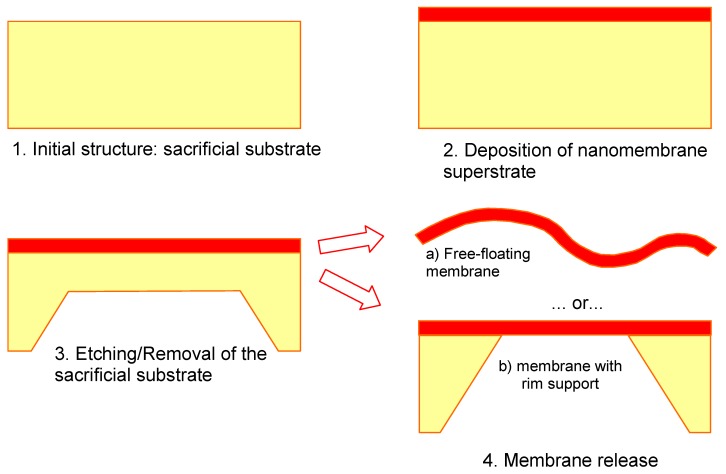
Generic technological procedure for the fabrication of nanomembranes utilizing sacrificial support.

Figure 1 shows the technological steps for nanomembrane formation on a solid sacrificial support. Many methods differ among themselves only in the deposition step (2 in Figure 1) and the number of methods for thin film fabrication in this step, both top-down and bottom up, is virtually limitless. Some of the top-down methods used include RF or DC sputtering, electron beam technique, evaporation, electroplating, epitaxy, drop-evaporation method, various versions of chemical or physical vapor deposition, *etc.* Bottom-up methods include various self-assembly techniques.

It should be finally stressed that nanomembranes are extremely sensitive to ambient changes and their electronic, mechanical, optical and other properties are strongly modified by them (e.g., [22]). Even monatomic or monomolecular adhesion will cause relatively large modification of the overall characteristics. This is a logical consequence of the fact that nanomembranes are quasi-2D objects, thus having very high surface to volume ratio. In that way a very large portion of their constituent atoms/molecules is exposed to the influence of the environment and the surface effects are thus large. On one side, this is beneficial for the utilization of nanomembranes as sensitive elements in chemical or biological sensing, where the surface adsorption will cause a large change of the overall behavior. On the other side, this means that the nanomembrane properties will be easily modified by even minuscule changes in the surrounding species. This dictates a strict care when assessing the results of experimental measurements of nanomembranes and will probably result in conflicting reports of different authors with similar experimental setups. A careful control of the operating environments will thus surely be necessary. On the other hand, it mandates various methods for passivation of nanomembrane surfaces.

## 3. Nanomembrane Functionalization

In most of the situations, functionalization of simple nanomembranes is equal to their nanocompositing. This means the introduction of two or more phases, each contributing its desirable properties, to obtain a superstructure with pre-designed multifunctionality. The dimensions of the introduced phases will obviously have to be sufficiently small to fit into such obtained low-dimensional nanocomposite which itself has a nanometric thickness. Thus multi-functionalization of nanomembranes may be viewed as the extreme case of thin-film nanocompositing.

It is obviously expected that the effective parameters of a composite nanomembrane are superior to those of each of the constituent phases separately. Besides combining and enhancing the existing properties of the constituents, in certain situations completely novel effects and functions appear. A good example is electromagnetic optics of membranes with ordered arrays of pores. Instead of only modifying the transmission and reflection, such structuring introduces the novel effects, even the quite exotic and unexpected ones like negative refractive index or extraordinary optical transmission (super-transmission) [23], none of which have been observed in nature ever before.

The list of the functionalities that can be introduced to nanomembranes is vast. One could enhance the mechanical properties like hardness, elasticity, wear, tribological properties (typically lowering of friction). Wettability may be decreased or increased by surface corrugations or patterning. Electric conductivity may be increased or decreased, magnetic properties imparted, plasmonic behavior introduced or modified. Thermal behavior may be tailored (e.g., extreme values of thermal conduction, etc), as well as chemical activity. Optical and generally electromagnetic properties may be modified, including changes of the effective refractive index in vast range, introduction of antireflection properties, introduction and improvement of photoluminescence, tailoring of dielectric permittivity and magnetic permeability, *etc.* Biological functionalization may be also done (biocompatibility, attraction or repellence toward certain specific agents, *etc.*). This is obviously only a portion of available functions and one could say that practically any parameter may be modified and introduced or enhanced, as well as almost any desired combination.

Phases in a nanomembrane may be separated along the film thickness (lamination), be distributed within a single stratum (granular nanocompositing) or only on its surfaces (patterning, which itself may be additive or subtractive). A special case with a large importance is the introduction of pores into the nanomembrane. Besides changing the structure and composition, one may impart functionalization by changing the geometry of the nanomembrane, for instance making surface corrugation (sculpturing).

In the subsequent text we handle in more detail each of the applicable approaches to nanomembrane functionalization.

## 4. Layering/Lamination

The simplest method to functionalize nanomembranes is to fabricate sandwich structures, *i.e.,* to deposit ultrathin layers with different properties. The properties of each layer itself are not modified and the nanocompositing is obtained only through the combination of new planes. The newly added strata are still quasi-2D, *i.e.,* their thickness is orders of magnitude smaller than their width and length and is in nanometer range. The bottom limit to the thickness of a specific stratum is determined as that of a single monatomic/monomolecular layer. The obtained sandwich superstructures are variably denoted as layered, laminar or stratified. The layered nanomembranes are a special case of laminar nanocomposites.

The layered nanomembranes may consist of two strata (the simplest case) or more. Each stratum introduces its own properties and functionalities to the nanocomposite to contribute to its multifunctionality. For instance, one stratum may have a biological or chemical activity, e.g., a ligand layer to attract selectively only a specific species; another may be a plasmonic waveguide and ensure the possibility for biological sensing; the next layer may be introduced to ensure mechanical strength and robustness of the whole structure.

**Figure 2:**
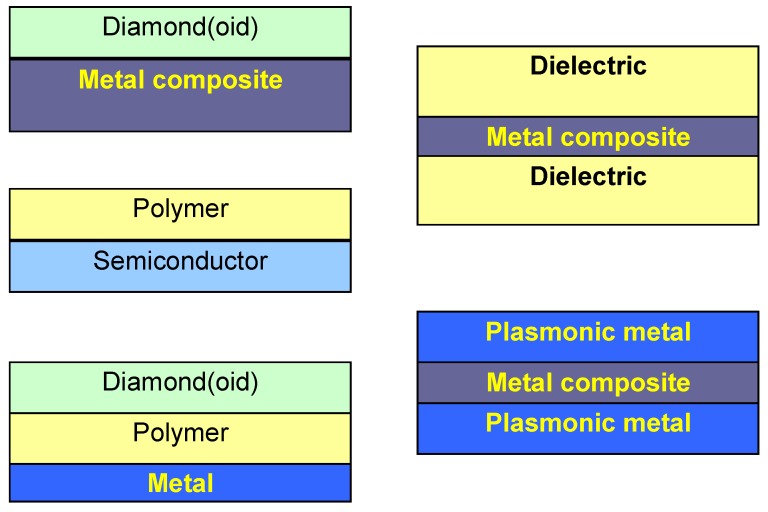
Several possible basic kinds of nanomembrane functionalization through lamination. Metal composite and/or polymer serve as ultrathin, freestanding support.

Figure 2 shows some basic configurations for laminar nanomembranes. The number of possible combinations of properties and functions is virtually limitless, and the basic restriction stems from the adhesion and possible reactions between different strata, as well as from the possible fabrication technologies.

The kinds of lamination are as numerous as are the required functionalities. Some of them may serve as a mechanical scaffold and support; such structures are indispensable if for instance extremely thin organic structures are required which cannot support themselves. These structures include diamond-like materials, carbon nanotubes, some semiconductors like silicon nitride, *etc.* Interfacial strata may be used for surface passivation. This is indispensable for chemically unstable surfaces, for reactive, oxidizable, *etc.* layers. Again, one may use diamond-like materials, but also polymers, metals, semiconductors and their combinations. Chemical modification may also be done by surfactants.

A special kind of multifunctional agents are those used to tailor electromagnetic properties: to increase or decrease optical absorption for instance, to tailor reflectance or to impart plasmonic functions. Generally, Drude metals (Ag, Au) can be used both to introduce plasmonic behavior and to increase reflectance [24]. Some metal oxides like tin oxide, indium oxide and indium tin oxide introduce at the same time low absorption and plasmonic behavior in the near infrared.

### 4.1. Microfabrication

The basic way to fabricate laminar nanomembranes is microfabrication. Practically all of the methods for thin film deposition are available to this purpose, the limits being set by the sensitivity of the particular materials to the operating conditions of the necessary technological cycles. Maybe the most often used technique is sputtering deposition of the desired material combination. Also available are vacuum evaporation and various kinds of vapor deposition – chemical (CVD) or physical (PVD). Epitaxial growth from vapor or liquid phase ensures fabrication of single crystalline layers, and among the applicable methods the molecular beam epitaxy surely stands out.

Other methods are available for more sensitive nanomembranes, for instance the organic ones, although of course these methods can be applied for the more enduring ones as well. One of the approaches to use spin coating [2], which utilizes the standard spinners for resist deposition, but the composition of the solution is determined by the desired material and required final thickness. Another convenient approach for sensitive multilayers is to use electrochemical deposition. Other conventional microfabrication techniques for such materials include dip coating, chemical deposition and others.

Two methods are especially convenient for nanomembrane layering and will be thus handled here in more detail. These are Langmuir-Blodget method and layer-by-layer technique.

### 4.2. Langmuir-Blodget Technique

**Figure 3:**
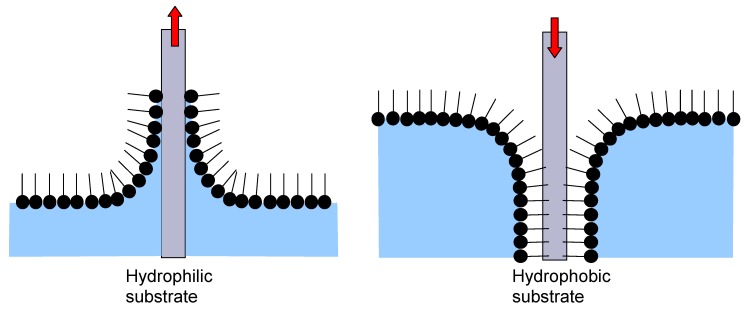
Schematic presentation of Langmuir-Blodget Technique for hydrophilic and hydrophobic substrates.

The Langmuir-Blodget (LB) technique [5,25] is the fabrication of lamellar nanofilms using amphiphilic molecules/surfactants like fatty acids, phospholipids, and glycolypids. When such molecules are placed at an air/water interface a monomolecular film is formed (a Langmuir monolayer) since the amphiphilic molecules orient themselves in such a manner to minimize free energy and thus form an insoluble monolayer. This monolayer can be transferred to a solid substrate utilizing its vertical movement into (immersion, dipping) or from (emmersion) the interface between the Langmuir monolayer and water (Figure 3). The whole procedure is repeated until the desired lamellar multilayer is formed.

The LB technique is a biomimetic self-assembly procedure, miming the forming of cell membranes and applying the process for the formation of multilayers. Various types of substrates may be used, either hydrophobic or hydrophilic. The method gives layers homogeneous over very large surfaces, enabling at the same time a thickness control at single nanometer level. The obtained layers may be further functionalized through incorporation of various macromolecules with specific activities.

An example of freestanding laminar nanomembranes fabricated by the Langmuir-Blodget technique can be found in [26]. The freestanding multilayers were fabricated in *N*-dodecylacrylamide polymer with a minimum thickness of 3 nm (equal to a thickness of a single bilayer). The multilayers were deposited on a substrate with an already deposited sacrificial layer. After treating the structure in solution, the nanomembrane was simply peeled off. Up to 700 layers were deposited in this manner.

### 4.3. Layer-by-Layer Technique

**Figure 4:**
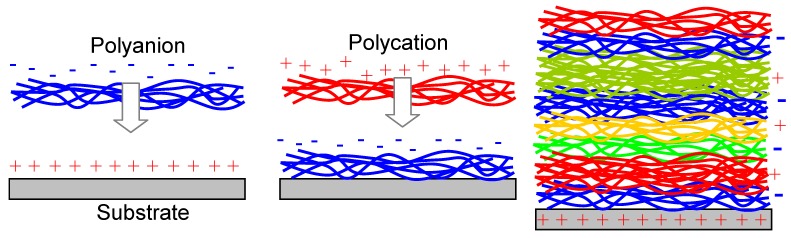
Layer-by-layer nanomembrane assembly technique. Left: assembly of the first layer; middle: assembly of the second layer; right: final structure.

The Layer-by-Layer (LbL) technique is a self-assembly method that utilizes the alternate adsorption of oppositely charged macromolecules [27]. In short, it proceeds as follows (Figure 4): the sacrificial substrate, for instance silicon diaphragm, is immersed into a dilute solution of a cationic polyelectrolyte. The polyelectrolyte adsorbs as a single monomolecular sheet with a thickness of about 1 nm, which depends on the particular material used, after which the wafer is rinsed and dried. In the next step, the polycation-covered substrate is placed into a dilute dispersion of polyanions or negatively charged nanoparticles. A new monolayer is formed over the previously deposited one, and the wafer is again rinsed and dried. In this way a single cycle is finished of the self-assembly of a polyelectrolyte monolayer onto the substrate. Now the procedure may proceed in an analogous manner, utilizing the same material pair or a new, different set. 

Various molecules, proteins, enzymes, antigens and others materials can be assembled in this manner. The order can be pre-planned and the number of thus deposited strata may be very large. Structures 5–500 nm thick were formed in this manner. This technique is simple and does not require complex instrumentation. The LbL technique is sometimes called “molecular beaker epitaxy,” [28] meaning that by simple instruments and utilizing self-assembly one can produce molecularly organized films similar to the ones obtained with highly sophisticated and expensive technology of molecular beam epitaxy. 

The layer-by-layer technique is popular in fabricating freestanding nanomembranes and many different structures have been fabricated in that manner [1,12]. LbL structures may be very robust. For instance, LbL polymer nanomembrane laminated by clay flakes were produced, 100 nm × 1,000 nm and about 1 nm thick with a Young's modulus of about 270 GPa [29]

### 4.4. Simple Membrane Transfer

An approach to lamination of nanomembranes is membrane transfer, where a freestanding membrane is fabricated and simply placed over another one to adhere to it. Peng *et al.* fabricated single-crystal/amorphous multilayer nanomembranes utilizing this approach [30]. They used SOI wafers and applied HF solution to etch away the underlying silicon dioxide layer and free single crystalline Si nanomembranes. To accelerate the etching process and facilitate release, they fabricated arrays of square holes in silicon. Finally, they placed the nanomembrane over the final substrate where it adheres to its surface. Their bonding is enhanced through annealing at 500 °C. As the next step, they deposited a stratum of spin on glass onto the Si nanomembrane. The whole process of nanomembrane transfer and spin on glass deposition is repeated several times, thus creating a layered nanocomposite consisting of single-crystal semiconductor and amorphous insulator. Germanium nanomembranes on flexible substrates fabricated in a similar fashion were reported in [31]. Membranes containing both silicon and germanium were reported by Scott and Lagally [22].

**Figure 5:**
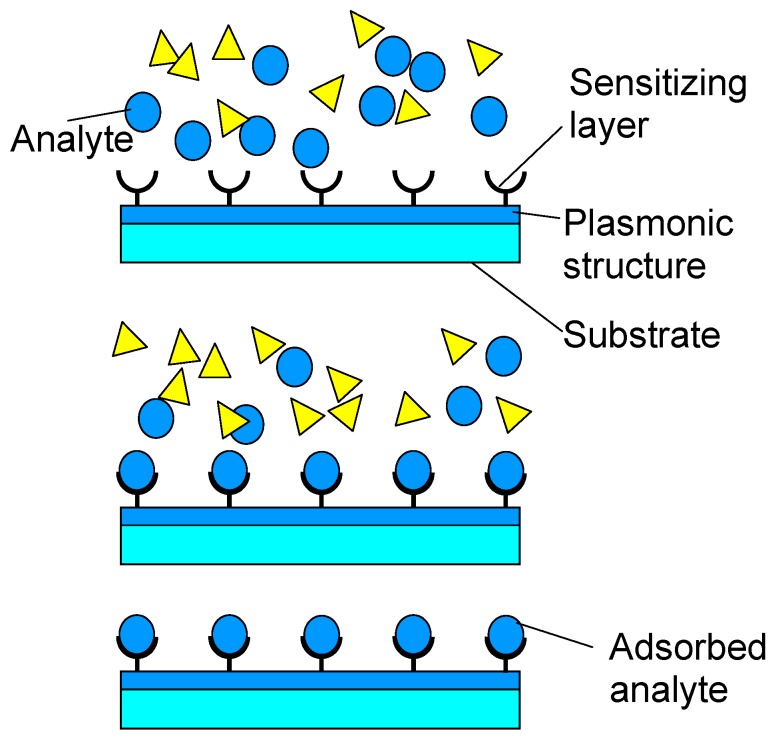
Surface sensitizing of long-range surface plasmon polariton sensors by depositing target-specific binding agent.

An important application of functionalization of nanomembranes through lamination is the deposition of sensitizing layers (ligands, enzymes, *etc.* binding the specific target analyte) to the surface of plasmonic sensors. It is known that long-range surface plasmon devices are the most sensitive contemporary plasmonic sensors [32]. The nanomembranes ensure a platform for that purpose with a right thickness (of the order of tens of nanometers) to ensure plasmon coupling on both sides and thus forming of long-range plasmons. At the same time, it ensures full electromagnetic symmetry of the structure, which is also convenient for long-range plasmon formation [24]. The deposition of binding layers on both surfaces of the nanomembrane ensures the selectivity of thus obtained sensors (Figure 5). A method to deposit sensitizing layers to the nanomembrane is the described layer-by-layer technique.

## 5. Surface Activation

The surface activation and generally surface modification includes all the various methods for the application of external means in order to alter the boundary/interfacial atoms or molecules of the nanomembrane that come in direct contact with the environment. These methods may involve breaking bonds at the interface or creating/re-creating new ones, generating e.g., polar groups, removing or modifying boundary atoms/molecules, creating new links, *etc.* Such technique may drastically change the way the nanomembrane behaves and modify its parameters even for several orders of magnitude, although the activation does not enter more than a single monolayer into the nanomembrane - a somewhat expected behavior, since very often the interfacial region is what actually defines the nanomembrane response to external stimuli. 

In a certain sense the surface activation methods may be regarded as a subset of the lamination methods since the modified interfacial monolayer with new properties may be understood as a new stratum added or, more often, swapped with an existing one.

The methods for surface activation may be divided into several distinct groups. Some of them modify strictly the interfacial layer, while others may also cause changes throughout the nanomembrane. The chemical and electrochemical methods of surface treatment are probably the simplest and most readily applied. Plasma processing methods form another large group. These methods are among the most often used ones in nanotechnologies. Next are treatments with electromagnetic radiation. The subgroups include treatment with radiofrequent or microwave radiation, optical methods which include the application of infrared, visible and ultraviolet radiation. 

Among the methods that simultaneously modify the surface and the whole structure is irradiation with ionizing electromagnetic waves, which includes extreme ultraviolet, x-rays and gamma rays. Among the irradiation methods utilizing particles are bombardment with nuclear particles (alpha and beta particles, neutrons and protons, accelerator particles). Of the quoted methods, probably the most well known and widespread method for surface activation is plasma functionalization. It has been used both for the activation of surfaces of conventional membranes and nanomembranes [33,34].

The application of ionizing radiation to nanomaterials modification was considered in [35]. The extent and the consequences of the nanomembrane surface modification are very clearly seen in [36]. The band structure of silicon nanomembrane was changed by chemical activation of the membrane surface using hydrofluoric acid and the result was the change of the semiconductor type and a large increase of conductivity. To this purpose the surface of 27 nm thin Si nanomembrane layers with 10^15^ cm^-3^ p-type doping were treated by hydrofluoric acid. This moved the Fermi level closer to the conduction band minimum. The result was an inversion of the starting p-type silicon to n-type nanomembrane and consequently a large drop in sheet resistance. This is in contrast with bulk silicon and is caused by the fact that in the nanomembrane the interface trap density in the gap will exceed the sheet density of dopants. Activation of silicon and diamond surfaces in order to optimize them for biosensing applications was considered in [37].

## 6. Patterning

A nanomembrane may be functionalized by motifs or shapes organized in a regular 2D arrangement on its surface(s). Such a pattern may be additive or subtractive (the regular motif may be added onto the surface or the membrane material itself may be partially removed to create a complementary arrangement. Examples of additive nanopatterning for multi-functionalization include e.g., depositing islands of quantum dots, islands of plasmonic nanoparticles, *etc.* Since nanomembranes are quasi-2D, removing material from them usually equals making a hole through the whole structure, *i.e.,* making a pore. Thus in this Section only additive patterning is considered, while subtractive is covered in the section on Pore Forming.

The added material will be typically different from the membrane material itself, albeit patterning with the same material may also be desired to increase multifunctionality, for instance to improve selectivity of membrane material towards certain chemical substances and biomaterial. 

The additive methods of functionalization may actually be seen as a special case of the lamination, where only a partial coverage of the nanomembrane surface is done, contrasted to the standard lamination/layering, where the coverage is 100%. This may be done by first covering the whole surface and then removing a part of the deposited layer, or directly depositing only the patterning element. The patterning itself may be done in one or more layers. The deposition process may be done onto the released nanomembrane or prior the releasing process, onto the nanomembrane precursor on sacrificial substrate.

A patterning procedure is usually preceded by the formation of a template for the patterning, which may be produced using various top-down and bottom-up approaches. It should be stressed that the template formation process is applicable to the additive and subtractive patterning procedures, the difference being in the subsequent steps, which may either add or remove material.

### 6.1. MEMS Patterning

Many methods belonging to the top-down techniques for microsystem and nanotechnology fabrication can be used for patterning prior the nanomembrane release from its support [21]. The nanomembrane precursor on the sacrificial substrate is much more robust than the released nanomembrane and in principle can withstand chemical, mechanical and temperature treatments better than freestanding structures.

Conventional photolithographic masks can be used to define patterns and any of the microfabrication depositing techniques (RF or DC sputtering, vacuum evaporation, some of the vapor deposition methods like CVD, local epitaxy, chemical and electrochemical deposition, *etc.*) may be applied. Direct writing techniques are also applicable. As far as the size of the patterns is concerned, for sufficiently large ones (near micrometer and larger) the conventional UV lithography is sufficient. To form nano-patterns one has to use extreme UV lithography, e-beam, FIB, x-ray lithography, microcontact printing/nanoimprint, scanning probe lithography and similar methods [21]

In case when biological and generally more sensitive materials are used, one usually must avoid techniques requiring vacuum deposition and elevated temperatures, as well as aggressive chemicals. In that situation the fabrication toolbox significantly shrinks and reduces mostly to methods of chemical and electrochemical deposition. Soft lithography was also applied, which uses poly(dimethylsiloxane) elastomer as a stamp or a mold. Various modified imprint methods were used. 

**Figure 6:**
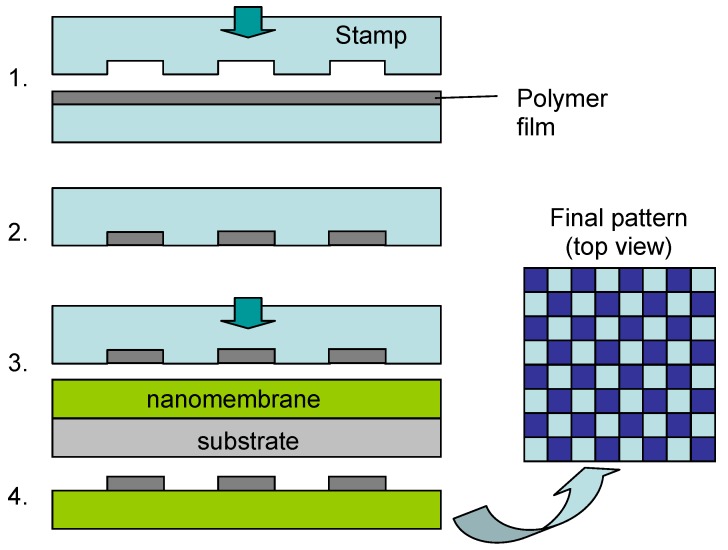
Patterning utilizing capillary transfer lithography. 1. Pattern transfer to a stamp; 2. Released mold; 3. Transfer to a nanomembrane on a substrate; 4. Patterned nanomembrane released from the substrate.

Ko *et al.* [38] utilized capillary transfer lithography to fabricate chessboard and striped arrays of single layers of carbon nanotubes and gold nanoparticles. To this purpose, they used a variant of the soft lithography technique, which they termed capillary transfer lithography. The filling is based on capillary principle and the pattern is transferred using mold (Figure 6). The structures were transferred to LbL nanomembrane precursors.

Another proposed approach was to apply photolithography directly to layer-by-layer films [38]. In that manner, freestanding nanomembrane structures were formed and used to form ultrathin cantilevers.

An interesting case of additive multilayer patterning, which also has 3D corrugation, was described by Kawakami [39] as the autocloning technique. In their experiments, a patterned layer was formed, to be subsequently sputtered over by continuous sheets. The 3D geometry of the first patterned layer was further transferred – "cloned" to subsequent layers, so that full 3D structures were formed. This approach was utilized to fabricate photonic crystal surfaces.

### 6.2. Self-Assembly Methods

The self-assembly methods offer a bottom-up approach to creating topography on the nanomembrane surface where the self-organized pattern is replicated in the chosen material. They offer some conveniences hard to achieve by alternative methods. They enable fabrication of ordered features with periodicity starting at nanometer dimensions. At the same time, they can extend over large areas of millimeters square, even centimeters square, thus furnishing giant aspect ratios. Neither direct writing methods nor high-resolution lithography can easily furnish this. Further, self-assembly methods are convenient for work with sensitive structures, including organic and even biological components, since they are typically implemented at room temperature. Actually, the possibility to use the organic and even biological materials significantly expands the toolbox for the nanopatterning of nanomembranes.

Typically the material for self-assembly is deposited utilizing some of the methods which are compatible both with the nanomembrane and the material to be patterned. This may be deposition from a solution, e.g., by spin coating. The material is left to self-assemble into ordered patterns on the nanomembrane precursor surface, a functionalizing material is added by a compatible micro/nanofabrication technique, the templating agent is removed and the nanomembrane is finally freed.

An important method for self-organization of patterns on nanomembranes is the use of block-copolymers [40]. Depending on the kind, these materials may form numerous different nanopatterns. Typical periodicity in self-assembled block-copolymer varies in a range from about 5 nm to several hundreds of nanometers. The periodicity length of the block copolymer patterns is directly determined by the molecular weight of the chosen material, which means that the unit cell of the obtained pattern is tailorable through the choice of the block copolymer.

The templating procedure using block copolymers proceeds as follows. First, the chosen block copolymer is deposited from solvent using e.g., spin coating. The next step is self-assembly of the block copolymer, followed by the removal of one of the copolymer blocks. This leaves the nanomembrane precursor substrate coated with a structure of ordered voids. The next procedure is the patterning process which proceeds through the mask formed during the self-assembly and material removal steps. It is possible to use MEMS/nanotech deposition methods to form additive patterns utilizing metals, semiconductors, oxides, magnetic, plasmonic, organic materials. One may use evaporation, sputtering, chemical vapor deposition, *etc.*, may deposit material chemically or grow it electrochemically. Finally, block copolymer is fully removed using some of the methods like solving, reactive ion etching, ashing, *etc.*, leaving the deposited patterns on the nanomembrane precursor.

Another important group of templating materials includes liquid crystals. The characteristic length scales are smaller than in the case of block copolymers and are of the order of 1–10 nm. Very large areas can be covered. Typically the lyotropic liquid crystals are used [41,42] which contain amphiphile molecules (having hydrophobic and hydrophilic parts, similar to phospholipids from the living cell walls) plus solvent. This results in a very complex and rich behavior in water, giving a number of different self-organized forms. Periodic structures with cubic symmetry are available, hexagonal, lamellar, bicontinuous).

There are two approaches to templating using liquid crystals. The liquid crystal nano-templating generally is done using nonionic amphiphile. One of the procedures is direct templating, where the liquid crystal is self-organized whilst containing precursor of the material to be formed (e.g., semiconductor, metal, *etc.*). After templating, the desired material itself is introduced. Another is conventional templating, where liquid crystal precursor is assembled on the nanomembrane, the deposition of the desired material is performed (however, due to sensitivity of liquid crystal materials, no procedures requiring vacuum or high temperatures are applicable and the best choice remain chemical and electrochemical approaches).

Patterning of nanomembranes may be also done by colloidal templating. To this purpose, silica or polymer spheres in colloid are used. When settling, they self-organize into a cubic lattice. Minimum features available by this method are of the order 10–20 nm, although features down to several nanometers are in principle possible. The method was introduced for the fabrication of 3D photonic crystals and most of the experiments were thus done at larger length scales, of the order of a hundreds of nanometers.

The obtained structures are denoted as synthetic opals [43]. Also complementary structures were fabricated, denoted as inverse opals, by imbibing the synthetic opal template using some of the techniques like sol-gel method, introduction of nanoparticles, melts or solutions, chemical vapor phase deposition, *etc.* and then etching away the spheres.

It was already mentioned that work on nanomembrane is often biomimetic, or more precisely bio-inspired. One of the approaches to nanomembrane patterning related to biology is the use of the nanoporous bacterial S-layer as a templating material [44]. In this method cadmium sulfide lattices were formed with a form directly copying the S-layer structure.

### 6.3. Direct Patterning of Freestanding Nanomembranes

The most difficult method is to pattern directly the freestanding nanomembrane. Very careful approach must be chosen in order to avoid damages to these ultrathin structures, since heat, chemical ingredients and mechanical handling can easily damage them. From this reason, this technique is seldom used.

One of the approaches is to use modified implementation of the conventional microfabrication techniques in order to avoid inflicting damages. A path toward this is to utilize self-ordering of materials deposited by some of the conventional methods.

Kim-Lee *et al.* [45] deposited Si_0.36_Ge_0.64_ on both sides of a 23 nm thick freestanding nanomembrane utilizing chemical vapor deposition. In this way they produced 3D SiGe "hut" islands 70 nm thick, which heavily stressed the nanomembrane. They observed self-ordering of the islands and explained it by the appearance of a strain field due to the initial island growth and the subsequent guiding of nucleation of subsequent islands on the opposite surface.

## 7. Pore Forming

Fabrication of pores with characteristic dimensions of the order of nanometers may be the most important kind of functionalization not only for nanomembranes, but also for membranes generally. Many critical applications depend on them, including those in filtering and separation.

**Figure 7:**
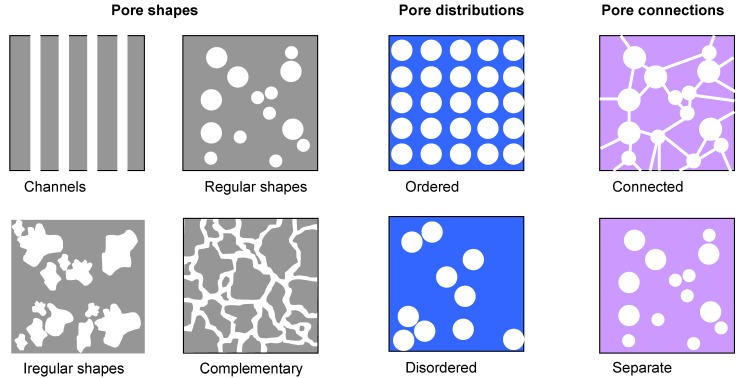
Some possible pore shapes, distributions and connections.

Voids in material may be regarded one of its two constituent phases, the other one being the basic material, thus a porous structure may be regarded a special kind of nanocomposite [46]. Tailoring of the structure of pores requires control over two main parameters. One is the desired pore geometry (shape and size) and the desired distribution. Regarding the shape, pores may be somewhat arbitrarily classified into several groups: linear pores which have one dimension much larger than the other two and may be straight (actually nanochannels going throughout the structure) or bending; equi-axed in which all three dimensions are of comparable size and which may have regular shapes (spherical, ellipsoidal, etc) or irregular. A sample may have one or more characteristic pore shapes. Pores may be separate or continuous (connected) throughout the structure (Figure 7).

As far as the dimensions of the pores are concerned, it is necessary to make a distinction between different nomenclatures regarding this matter, since it may be a source of some confusion. According to the IUPAC, the term macropores denotes pores larger than 50 nm, mesopores are between 2 nm and 50 nm and micropores have dimensions below 2 nm [47]. In some literature sources all pores with diameters below 100 nm are simply denoted as nanopores (e.g., [48]). The size of pores within a single structure may be comparable or may significantly vary. Finally, the distribution of the pores themselves within the structure may be ordered or disordered.

A special case of porous nanomembrane are those with straight or curved pores going all the way from one side to the other (channel type); their distribution across the membrane surface may be disordered or patterned. In the latter case, they belong to the previously mentioned subtractive patterning. Still, for the sake of completeness and because of the importance of the pore formation as a separate field, they are handled in this Section.

As mentioned before in this text, a large number of micro/nanofabrication techniques cannot be utilized for the fabrication of pores because of the sensitivity of the nanomembrane themselves. No high energies and excessive heat must be used. This is especially valid for the fabrication of pores within already released membranes, while the processing of the structures still in their precursor stage and thus protected by their substrate leaves a greater degree of freedom - although in the later stages presents a problem during the substrate removal and nanomembrane release. It is important to note here that when fabricating nanochannel pores through the structure one obviously has available all of the methods described in the section on additive patterning.

In this section we present the main methods for the pore fabrication in nanomembranes. We describe separately the methods utilizing directed energy or directed particles for pore forming and those utilizing direct synthesis of the pores. We stress the methods utilized for the fabrication of pores in already formed and released nanomembranes.

### 7.1. Directed Energy and Directed Particles

A system of pores can be fabricated in a nanomembrane by of a homogeneous starting membrane material either by high-energy particles or by directed radiation. When passing through the membrane these particles or beams form tracks. Pores can be formed either directly by evaporation of the material on the path of the beam or by subsequent etching. The authors of [49] denote such porous structures as track membranes. The desired shape and dimension of the pores can be defined either by lithography using a contactless mask or by direct writing.

Directed energy methods include those utilizing ionizing electromagnetic radiation – typically X-rays (frequencies 3 × 10^16^ Hz to 3 × 10^19^ Hz, *i.e.,* energies 120 eV to 120 keV). Also among the used sources is synchrotron radiation. Directed particles may be focused electrons and ions. Irradiation by nuclear particles is also sometimes mentioned and includes bombardment with alpha and beta particles and neutron bombardment

One could pattern nanomembranes by directed energy (X-ray and synchrotron radiation). To this purpose, one may exclusively apply lithography utilizing contactless mask, since the delicate nature of nanomembranes precludes the use of contact methods. More convenient are direct write methods utilizing accelerated charged particles, including e-beam and focused ion beam (FIB).

The directed energy and directed particle techniques are applicable both to nanomembrane precursors on a substrate and to released freestanding nanomembranes. Their application on the nanomembrane precursor follows the usual route for the micro/nanofabrication techniques [50]. Thus it will not be considered in this Subsection. We will focus instead to direct patterning of freestanding structures after they are fully released, *i.e.,* the engraving process is not done for the ultrathin structures located on some kind of a substrate.

The method of choice for the fabrication of ordered arrays of pores in nanomembranes is the FIB technique, due to its short penetration and interaction ranges. In the case of freestanding nanomembranes, the interaction of accelerated ions occurs with structures thinner than the projected ion penetration depth or comparable to it. It is convenient if the nanomembrane is homogeneous and if should be electrically conductive (in order to dissipate the electric charge brought by the incident ions).

The physics of the ion collision with thin metal sheets (gold) was analyzed in [51], and that of ion interaction with SiC nanomembranes in [52]. At sufficiently low energies, ions interact with the nanomembrane material in a series of binary collisions ("a collision cascade"). With increasing energy, the mean free path of ions between two successive collisions approaches the interatomic spacing of the nanomembrane. In that case, the interaction cannot be approximated by independent binary collisions, since the successive collision "feels" the previous one. Each collision forms a small perturbed region with increased energy (the so-called energy or displacement spike).

During several tens of picoseconds after the collision, the energy of the spike is shared more or less continuous by the atoms sufficiently near to the collision location. If the ion energy is sufficiently high, the effective temperature of the atoms within the zone will rise above melting.

Two limiting cases can be then discerned. For low ion energies and high flux densities, holes will be formed within the nanomembrane by its physical evaporation. For high ion energies, each ion impact will lead to a penetration through the whole membrane as a consequence of the atom displacement from the lattice, not because of evaporation. The resulting holes have diameters of several nanometers. For intermediary values of energies, both effects occur at the same time.

The exact levels of low and high energies depend on the nanomembrane thickness and composition; for metal-composite membranes the low energy limit is tens of keV, and high energies are of the order of hundreds of keV.

This means that too low ion energies are simply not sufficient to allow ion penetration through the nanomembrane and the formation of apertures, while too large energies tear and melt the nanomembrane (over-etching of the structure).

Gierak *et al.* demonstrated the fabrication of holes with diameters down to about 3 nm in silicon carbide (SiC) nanomembranes with a thickness of 20 nm [52]. Their experiments started from a silicon carbide nanomembrane with a thickness of 20 nm. A 35 keV Ga^+^ beam was used, focused by a 5 nm probe which transported 2 pA. The point dose within a single writing field varied continuously between 10 to 200 ms, which furnished fluxes of 2.5 × 10^6^ to 5 × 10^7^ ions per point. The obtained nanoholes were almost spherical, with diameters 2.5 nm to 4.5 nm, with a minimum diameter of 2.5 nm. The zone damaged by ion collisions around each nanohole had a width of about 15 nm.

**Figure 8:**
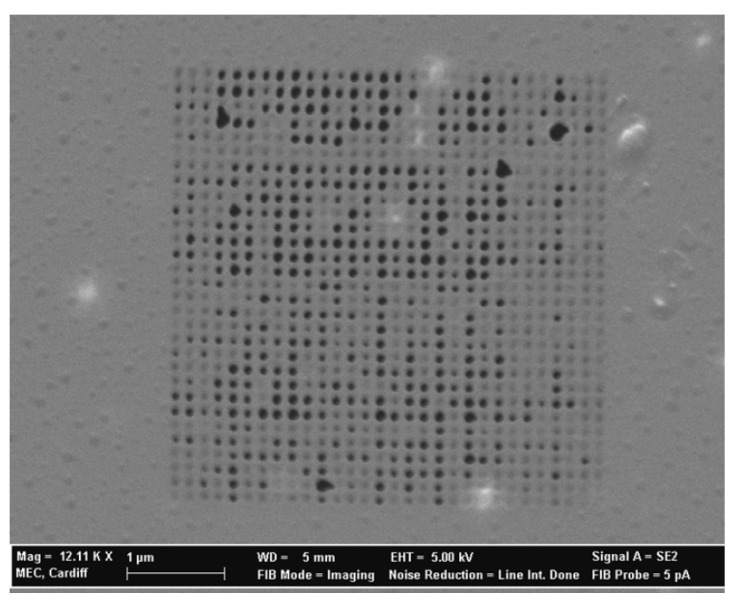
SEM image of pores formed directly in a freestanding metal-composite nanomembrane using the focused ion beam method. The hole diameter was about 50 nm, and the ion dose was 3.5 × 10^14^ ions/cm^2^).

Matovic *et al.* fabricated by FIB 2D arrays of nanoholes organized in square lattice pattern in 8 nm thick metal-composite Cr-containing nanomembranes with giant aspect ratio utilizing focused ion beam technique [53], Figure 8. The starting medium were metal-composite structures with approximately 50% Cr, 23% Si and 27% oxygen and a thickness of 8 nm (approximately 20 atomic layers), an area of 1 × 1 mm (an aspect ratio of 125.000). 30kV Ga^+^ ion beam was used to pattern an ordered 2D array of holes. The ion probe current was 5 pA, dwell time 0.02 ms, pixel step size 16 nm. Four scan loops were done over the processed area.

The optimum ion flux for the formation of patterned nanoholes in metal-composite nanomembrane was about 3.5 × 10^14^ ions/cm^2^, and the hole diameter was 42 nm. This is a much lower level of ion flux than used for ultrathin structures at solid substrates, since for bulk chromium-based materials such milling occurs for doses in excess of 5 × 10^16^ ions/cm^2^.

It is interesting to note that in these experiments no swelling of the nanomembrane material occurred, in contrast to the situation with bulk material, where Ga^+^ ions are implanted and the overall volume is thus increased. However, the nanomembrane thickness is so small that instead of being implanted gallium passes directly through its whole thickness, since the penetration range of the Ga^+^ with a beam energy of 30kV is greater than the nanomembrane thickness.

An interesting example of the FIB fabrication of nanometer-sized pores in ultrathin multilayers (laminar structures) can be found in [54]. An ordered matrix of square opening (the "fishnet" structure) was fabricated in structures with as much as 21 layers of silver and magnesium fluoride. In this way, the authors demonstrated a 3D metamaterial with negative refractive index in the optical wavelength range.

### 7.2. Direct Synthesis of Porous Structures

**Figure 9:**
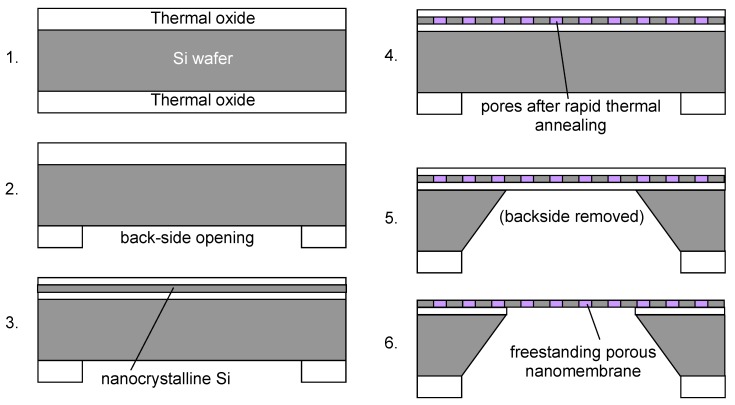
Fabrication of a porous Si nanomembrane by standard microfabrication methods according to [13].

An approach to fabricating porous structures within nanomembranes is direct synthesis of the nanomembrane precursor, followed by a treatment to generate pores. Striemer *et al.* performed that utilizing standard microfabrication method. They used rapid thermal annealing of deposited silicon [13]. Nanocrystalline silicon 15 nm thick is used for the nanomembrane precursor. The fabrication procedure proceeds as follows (Figure 9). Thermal oxide layers are first formed on both sides of a single crystalline silicon wafer. The backside layer is used as masking materials and openings for wafer silicon removal are etched in it. The top layer of the oxide is removed and a triple layer oxide-nanocrystalline silicon-oxide is deposited. The whole structure then undergoes rapid thermal annealing and its result is the pore formation within the Si layer. They use wet etching to remove the wafer silicon until only the oxide-Si-oxide layer is freestanding over the opening. Finally they remove oxide masks as well and the result is a freestanding, porous nanocrystalline nanomembrane. The average pore sizes ranged from 5 nm to 25 nm. The structure was used to nanofiltration of proteins.

Silicon-based polymer-derived ceramics [55] were used to fabricate microporous or mesoporous membranes on porous substrates. To this purpose sol-gel-derived preceramic polymers were utilized to form highly thermally and chemically stable porous membranes. Si-C-N membranes were fabricated starting from a poly(organosilyl)carbodiimide precursor film utilizing spin coating without solvent, followed by pyrolysis of the precursor in argon atmosphere at 1,000 °C [56]. SiBCN ceramic for high-temperature membranes was formed by hydroboration of 1,3,5-trivinyl-1,3,5-trimethyl-cyclotrisilazane with borane dimethylsulphide [57]. The polymer-to-ceramic transformation was again achieved at about 1,000 °C in argon atmosphere, and the membranes were finally formed by the dip-coating technique. Some of these membranes are applicable as molecular sieves, even for hydrogen molecules.

### 7.3. Artificial Ion Channels

A special type of structures in many ways connected with the pore complexes in nanomembranes are gated ion channels. These structures are ion-transmitting channels, and in nature they typically consist of proteins [58]. Their artificial counterparts in a general case may be fabricated in various organic and inorganic materials and their combination. 

An ion channel may be gated by an external stimulus whose presence or absence opens of closes the channel. A very often met gating mechanism is the applied voltage. Another one is the presence of ligands controlling the ion transport through the nanochannel. The transport of ions in both cases is highly selective. 

Various ion channels exist for different ions, for instance sodium, potassium, chloride, *etc.* This mechanism is fundamental for the ion transport through lipid bilayer nanomembranes in biological cells and represents the basis of the life on earth. 

As far as the fabrication of biomimetic pore complexes capable of selectively transporting various ions, there have been many experiments dedicated to this topic. Recently, an artificial scaffold for the nuclear pore complex-based gate was produced using natively disordered proteins termed FP nups. Such synthetic scaffolds can be used as a generic platform for ultra-selective biomimetic nanopores [59]. Another pathway is to avoid proteinaceous nanopores and to utilize other materials, including inorganic ones. Some convenient materials for such nanochannels include silicon, silicon nitride, polyethylene terephthalate, silicon dioxide and many others.

We consider here the ion channels enabling transport of protons (hydrogen ions) while at the same time blocking all other ions. In the living cells this occurs through the proton hopping mechanism (Grotthuss transport) [8,60]. In certain geometries of nanochannels water molecules align to form a chain (the quasi-1D “water wire”). In such systems, if the conditions are fulfilled, the proton hops from one water molecule to the next, while the water molecules within the water wire do not move themselves. The transport of protons involves only successive temporary rotations of water molecules participating in the "hopping", but there is no net translatory motion.

The flow of protons through a biological cell membrane is as fundamental to the cell metabolism as the flow of water, being coupled to the energy cycle that fuels the cell metabolism. It is advantageous for a cell to have separate transport of water (to maintain the internal pressure) and protons (necessary for cell energetic). The water balance within a cell is managed though aquaporin water channels [61]. Aquaporins are proteins with a hole along their middle which enable the flow of water molecules and at the same time are impermeable to protons, although these are much smaller than water molecules. Opposite to that, proton nanochannels are highly selective to protons (*i.e.,* hydrogen ions) but are impermeable to water molecules. Any significant imbalance in proton or water concentration immediately causes cell dysfunction or death. Because of that, the both classes of channels are actively gated by the cell control mechanisms, enabling control of protons concentrations and internal osmotic pressure inside the cytoplasm within narrow margins. These molecular assemblies were definitely the first proton transport structures, designed by the nature and found in practically all organisms. A comprehensive analysis of proton channels is presented in [62].

**Figure 10:**
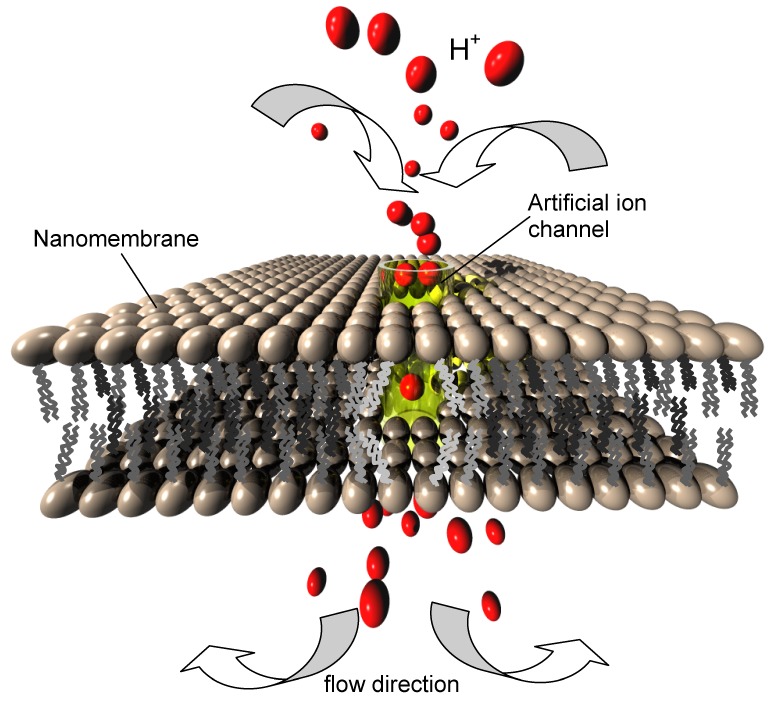
(a) Schematic presentation of an artificial gated ion channel through a phospholipid bilayer nanomembrane. The ion channel may be e.g., a carbon nanotube, a gramicidin structure, *etc.*

The proton conductive nanochannels may be gated or ungated. Probably the best known ungated proton channel is gramicidin A (gA), a small, double helix-shaped molecule with hydrophilic body and hydrophobic ends. Each helix contains a water wire and conducts protons via the Grotthuss mechanism. The proton conductivity of gramicidin channels is larger than in any other known ion channel and approaches 2 × 10^9^ protons per second. The incorporation of gramicidin A into the artificial phospholipid bilayer membranes is readily done [63], making synthetic proton-conductive structures (Figure 10).

**Figure 11:**
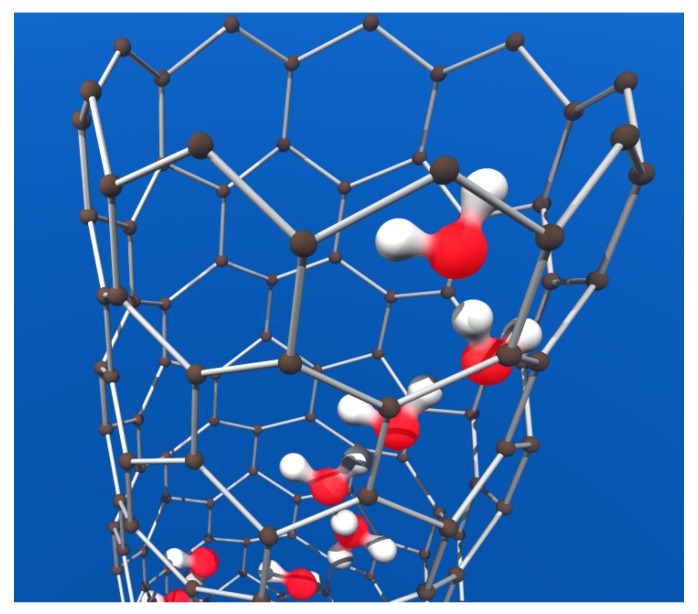
Carbon nanotube filled with water (red: oxygen, white: hydrogen, gray: carbon) forming Grotthuss-type proton conductive water wire.

Another possibility to fabricate artificial proton conductive nanochannels is to use water-filled single-walled carbon nanotubes, which already have built-in nanochannels (Figure 11). Compared to gramicidin A structures, they are far more robust, chemically stable and heat-resistant, which enormously extends the applicability of CNT-based proton conductors. The walls of CNT are hydrophobic, but can be made hydrophilic and filled with water wire [64], thus miming a gramicidin A structure. The third artificial structure proposed for synthetic proton-conductive channels are calixarene molecules [65].

Artificial incorporation of aquaporins or their artificial analogues, together with proton-conductive channels, could enable artificial nanomembranes with a full control of water/hydrogen flow, similar to the living structures.

## 8. Introduction of Nanofillers

In this part we consider the functionalization that may be done to a single stratum within the nanomembrane. In it, some kind of filler is introduced into the matrix, which is actually the simple nanomembrane to be functionalized. This approach actually represents granular nanocompositing of the membrane. Different phases are introduced into a single plane of the film and the result is a binary, ternary or higher composite. Most often, the phases are homogeneously and isotropically mixed, although the spatial distributions may be also gradient or anisotropic in another way. As in nanocompositing generally and actually in any compositing, it is expected that at the end the properties of the composite are superior to the properties of each of the constituents separately.

There are practically an infinite number of different shapes and forms to the phases to be introduced as functionalizing agent into the nanomembrane. Their sizes start below 1 nm and the limit to their thickness is the thickness of the nanomembrane itself.

**Figure 12:**
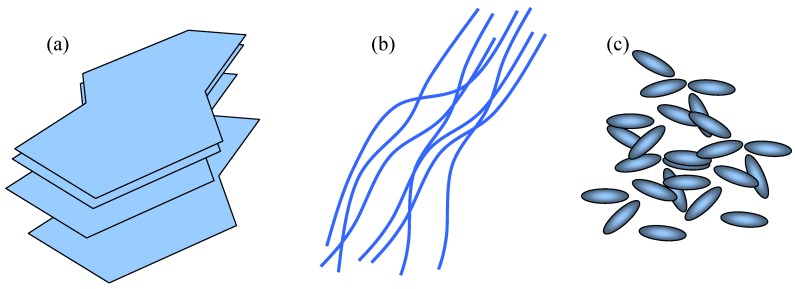
Classification of filler shapes for granular compositing of freestanding nanomembranes. (a) 2D fillers; (b) 1D fillers; (c) 0D fillers;

**Figure 13:**
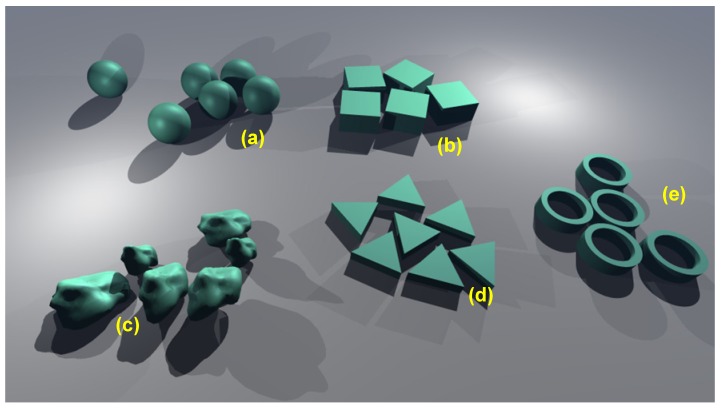
Some of many examples of equi-axed nanoparticles to be used as fillers for granular compositing of freestanding nanomembranes. (a) spheres (fabricated e.g., in silver, silica); (b) nano-cubes (silver); (c) irregular particles (silver); (d) triangular nanoprisms (gold); (e) nanorings (zinc oxide). The nano-building blocks are not to scale.

A general classification of filler shapes could be as follows (Figure 12):

(a) 2D fillers – plates or slabs where two characteristic dimensions (width and length) are much larger than the third one. Typical examples would be mica plates, graphene, various smectic clays (phosphosilicates), double hydroxides, dichalchogenide sheets, *etc.*
(b) 1D fillers – one dimension (length) is much larger than the other two and which includes various tubes, fibers and nanowires. Examples include carbon nanotubes, but also other types of nanotubes, for instance molybdenum and tungsten sulfides, vanadium and molybdenum oxides, *etc.*
(c) 0D (zero-dimensional) fillers – all three dimensions are very small. These are the nanoparticles in the strictest sense and they are more or less equi-axed. A plethora of different shapes belongs to this group. Some examples would include fullerenes, different Cd-based nanocrystals and actually the whole nano-"zoo" (Figure 13). The nanoparticles within the matrix may be ordered (organized, patterned) or may be disordered (stochastic – random distribution).

There are some peculiarities of nanofillers, which are a consequence of their size. Their low dimensionality and the resulting quantum effects impart them properties sometimes vastly different from those of bulk materials. From an electromagnetic and optical point of view, their strongly subwavelength size makes the nanomembrane containing them effectively homogeneous. They all have a large surface to volume ratio, which is useful in, for instance, catalytic reactions – and that property is often preserved after their inclusion into the nanomembrane matrix, since the nanomembrane themselves are quasi-2D and have a large surface to volume ratio. Mechanically, nanocomposite membranes may have an exceptional strength. This is because in a smaller filler particle a chance for a defect to appear is also smaller. The extreme situation is met with nanotubes and fullerenes, which are actually large molecules, and by definition should not have defects.

The nanofillers convenient for the functionalization of nanomembranes may be fabricated in a variety of ways. Different approaches utilize fabrication from solution (both aqueous and non-aqueous routes). One of the approaches is to use sonic bubbles (hydrodynamic cavitation). Another group of fabrication procedures includes aerosol methods, e.g., pyrolysis, flame hydrolysis, various condensation methods. As far as the inclusion of nanofillers into nanomembranes is concerned, this may be done by direct mixing of constituent materials, from a solution, or may be done in-situ during the nanofabrication of the nanomembrane precursor.

We consider here in some more detail the approach to the fabrication of metal-composite nanomembranes by Matovic and Jakšić [14]. It was used for the production of chromium-based nanocomposite freestanding nanomembranes with aspect ratios thickness to width/length approaching 1,000,000 and in a thickness range of 5–40 nm. The conventional microfabrication methodology was applied with somewhat unusual values of parameters in order to fabricate nanocomposite structures instead of the pure material membranes.

The starting material was Si wafer with <100> orientation. After wet oxidation, square windows were opened in the SiO_2_. KOH etching was used to fabricate square silicon diaphragms 15–20 μm thick. Each diaphragm had a silicon rim to serve as a support for the nanomembrane structure.

**Figure 14:**
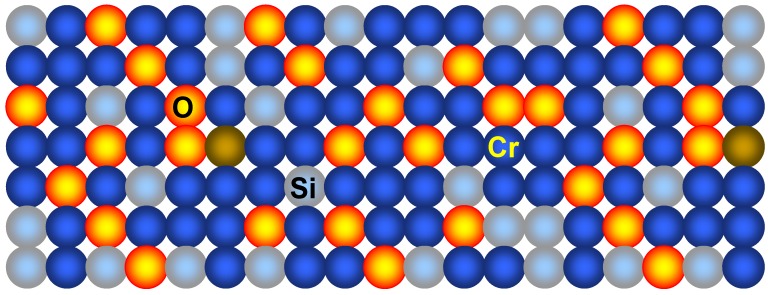
Cr-based metal-composite nanomembrane.

As the next step, the metal nanomembrane precursor in the form of a 5–20 nm chromium thin film was RF sputtered on the silicon wafer surface. To ensure the production of metal-composite structures and not pure metal layers some deviations were introduced compared to the standard MEMS procedures. The energy of particles was somewhat increased compared to the values customary in the art and was kept at about 5 eV. The result was that instead of the conventional Stransky-Krastanov growth chromium atoms had a sufficiently high kinetic energy to penetrate 0.7 nm, to the second atomic layer of the <100> Si wafer, without disturbing the crystal lattice. The obtained structure is schematically shown in Figure 14.

Another modification compared to the conventional approach to thin Cr film fabrication was that a certain controlled amount of reactive gas (for this process this may be oxygen or possibly nitrogen) was introduced into the system. The result was that some of the atoms deposited on the nanomembrane surface chemically reacted with reactive gas in the system to form an oxide or nitride (reactive sputtering). Further, since the total thickness of the deposited nanomembrane precursor was below 20 nm, the cathode surface did not perceptibly participate in the reaction. Because of that, the reactive sputtering process remained stable. Both lamellar and granular film growth occur simultaneously.

**Figure 15:**
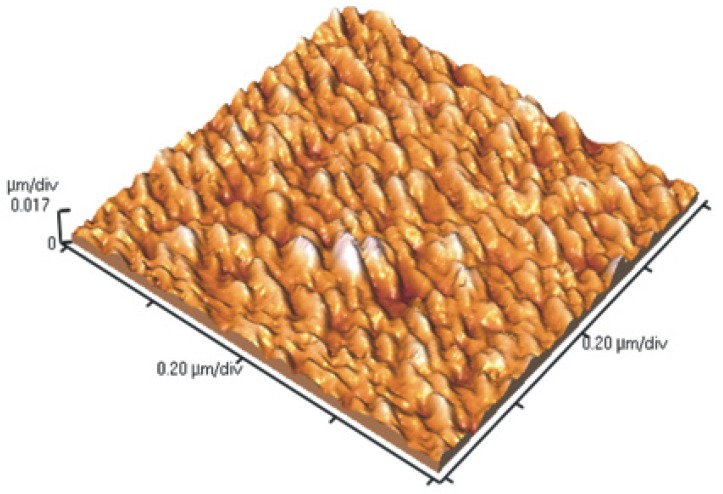
Structure of 7 nm thick metal-composite nanomembrane immediately after the removal from the etching solution. Corrugations are readily seen. After several hours of room-temperature self-annealing they completely disappear, leaving a surface flat within a few nanometers.

During the sacrificial silicon etching H^–^ ions are released and quickly neutralized by forming H_2_ molecules, which leave the solution as bubbles. Of these, a significant number of H^–^ ions diffuse directly through the silicon sacrificial membrane. When the H^–^ ions reach the deposited film, they are captured by the metal matrix and metal hydride is built. The volume of the metal hydride is much larger than the volume of the nanomembrane precursor itself and as a result, compression stresses build up. After the Si membrane is fully dissolved, the deposited film is released and becomes wrinkled while floating freely in the solution. This relaxed membrane can be removed from the solution without a fear of damages by capillary forces. Figure 15 shows an AFM image of a 7 nm thick metal-composite nanomembrane immediately after the removal from the etching solution. 

**Figure 16:**
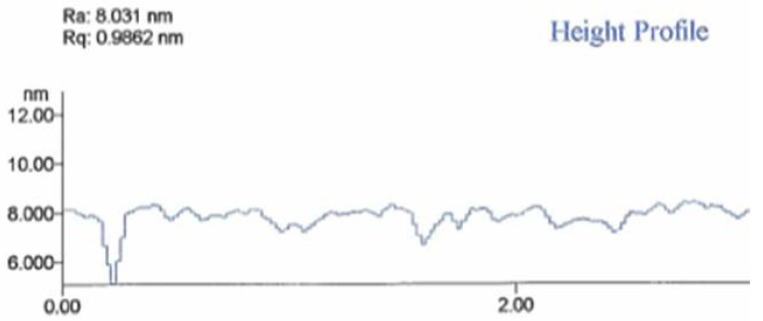
Height profile of 7 nm thick metal-composite nanomembrane surface.

The hydrogen captured in the metal hydride diffuses out after 1–2 hours. The result is that the nanomembrane precursor volume shrinks, creating a flat and smooth nanomembrane. This procedure actually represents a self-annealing process of the nanomembrane structure. A height profile of the nanomembrane after the self-annealing process as measured on a profilometre is shown in Figure 16, where it can be seen that its surface is smoothened within a range of about 1 to 2 nm.

**Figure 17:**
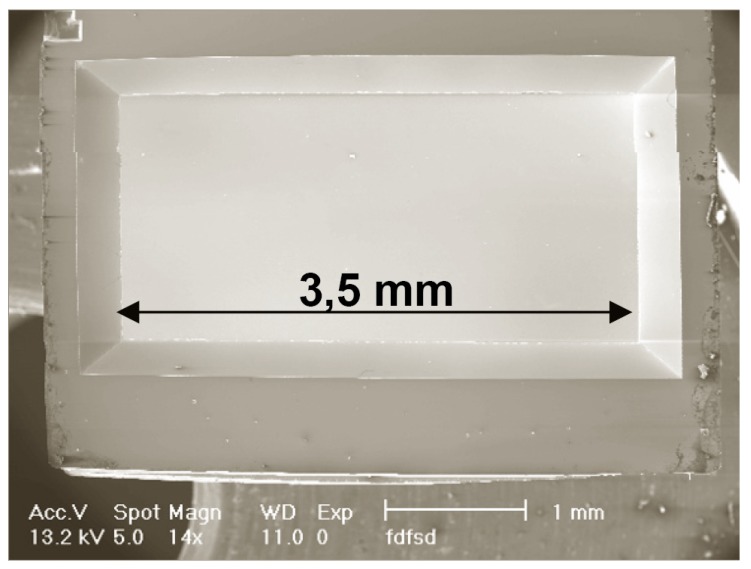
SEM image of a fabricated metal-composite nanomembrane, length 3.5 mm, thickness 8 nm.

**Figure 18:**
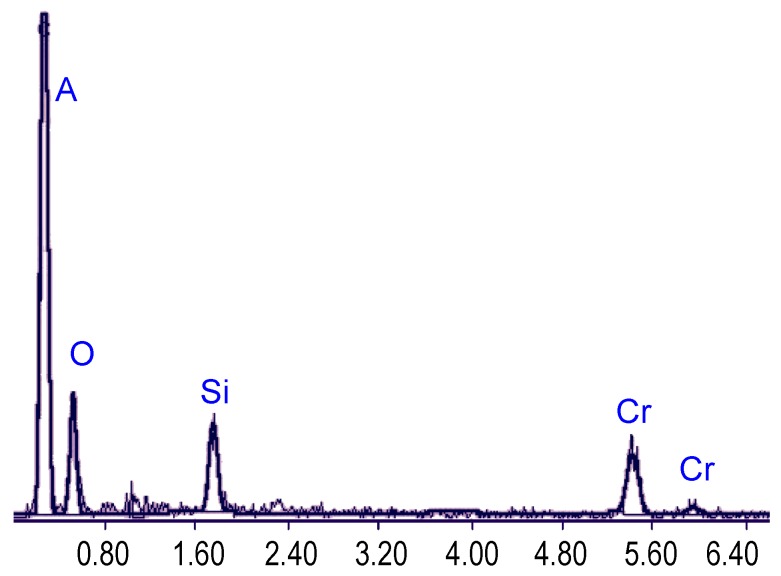
Energy dispersive X-ray spectrometry analysis of the nanomembrane composition: metal ~52%, oxygen ~28%, silicon ~ 20%.

A SEM image of a finished metal-composite nanomembrane after annealing is shown in Figure 17, and the diagram showing results of energy dispersive X-ray spectrometry analysis of the nanomembrane composition can be seen in Figure 18. The obtained composition is chromium approximately 52%, oxygen 28%, silicon 20%.

**Figure 19:**
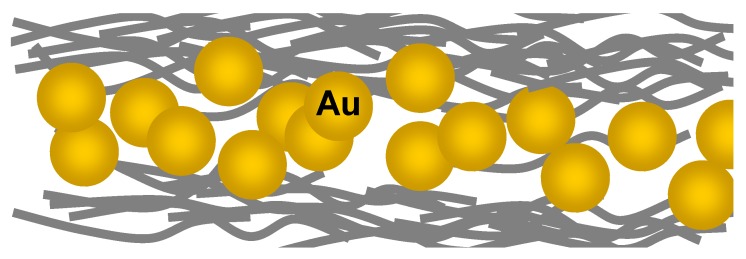
Polymer matrix (gray strands) with gold nanoparticle fillers; structures fabricated by Layer-by-Layer technique.

Other examples of ordered inclusion of nanofillers into nanomembranes are the structures fabricated by Jiang *et al*. [66] (Figure 19). They utilized a hybrid approach starting from the layer-by-layer membranes on sacrificial substrate and then applied to make organized patterns. As far as the fillers themselves are concerned, they deposited gold nanoparticles and carbon nanotubes. The layer-by-layer technique was then used again and finally the sacrificial Si was etched to release the freestanding nanomembrane.

Preparation of ultrathin nanofibrous freestanding films from the dispersions of cadmium hydroxide nanostrands was done in [67]. They used cadmium hydroxide nanostrands (1.9 nm in diameter, length of the order of micrometers) prepared from mixed aqueous solutions of cadmium chloride and aminoethanol. The cadmium hydroxide nanostrands are positively charged. They experimented with nanocomposites containing various other materials, including fullerenes or carbon nanotubes, negatively charged dyes, proteins and different nanoparticles with optical, biological, magnetic and plasmonic functionalities. Instead of using sacrificial single crystal, they used a polycarbonate membrane to filter the dispersion and then peeled of the freestanding composite nanomembrane.

## 9. Nanomembrane Sculpting: Tailoring Corrugations in 3D

**Figure 20:**
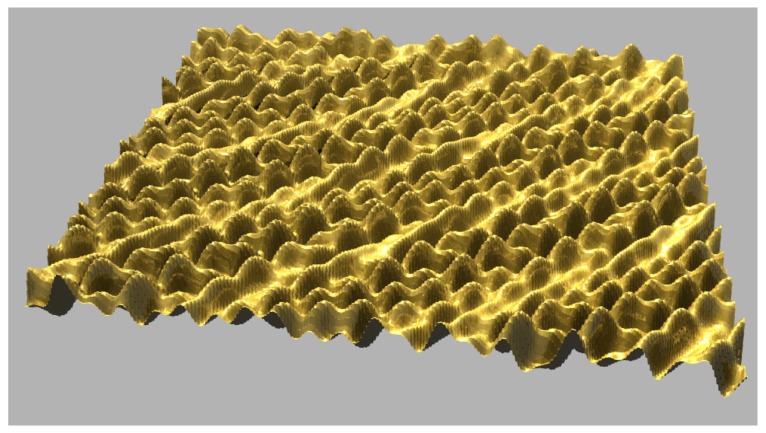
Wavy corrugations as 3D surface sculpting of freestanding nanomembrane.

A method to impart additional functionality to a freestanding nanomembrane film is to vary its topology, *i.e.,* to sculpt the film shape in the third dimension. Thus introduced surface modulations that take a form of ordered or disordered corrugations can be used to different purposes (see Figure 20). One of them is to change the optical properties of the nanomembrane. A corrugated structure will behave as a conventional or subwavelength dimensioned diffractive optical element. Such surfaces are utilized for instance as subwavelength anti-reflective structures; they are also applicable as couplers between propagating electromagnetic/optical waves and surface plasmons-polaritons. Other functionalities include tailoring of wettability (enhancement or suppression), selective adhesion and the improvement of biocompatibility.

A typical approach to topology tailoring of freestanding nanomembranes is to fabricate a template relief on the sacrificial substrate. The nanomembrane precursor is deposited over it and at the end of the process the template relief is etched away or the nanomembrane is peeled off. The 3D surface features remain in the released freestanding structure.

Lin *et al.* described the use of microstamping and sacrificial templates with their layer-by-layer films to fabricate wavy freestanding, self-supported structures intended to serve as subwavelength diffractive optical elements [68]. A sacrificial polystyrene plate was first micropatterned utilizing capillary transfer microprinting. The next step was layer-by-layer deposition of a 25-layer polymer film made of conjugated polymer MPS-PPV and poly(allylamine) hydrochloride (PAH). The substrate was finally dissolved and the sculptured surface was released as a freestanding nanomembrane. The thickness of their freestanding films was about 60 nm, the size of the patterns was several micrometers and the overall thickness of the corrugations (their peak-to-peak value) was 160 nm. The structures were intended for diffractive optics applications.

**Figure 21:**
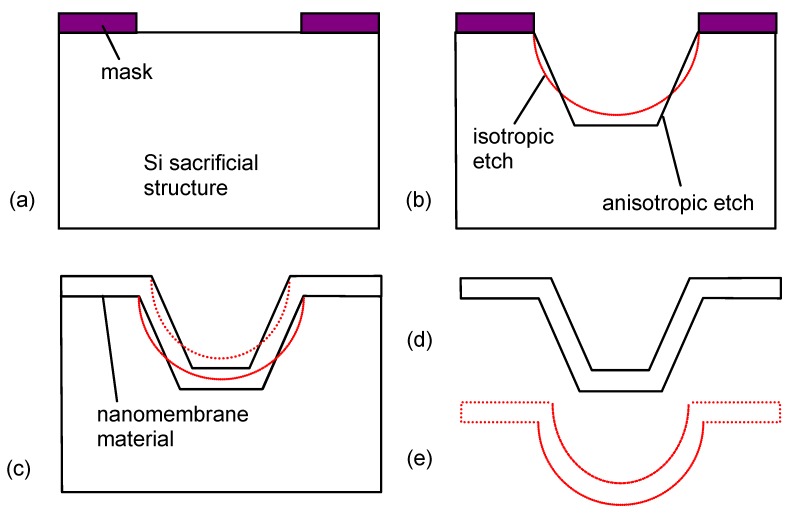
Technological steps for the surface sculpting of freestanding nanomembranes. (a) Si single crystal wafer with spinned-on mask; (b) etching; (c) mask removal and nanomembrane deposition; (d) sculpted structure after anisotropic etch of sacrificial silicon; (e) sculpted structure after isotropic etch.

**Figure 22:**
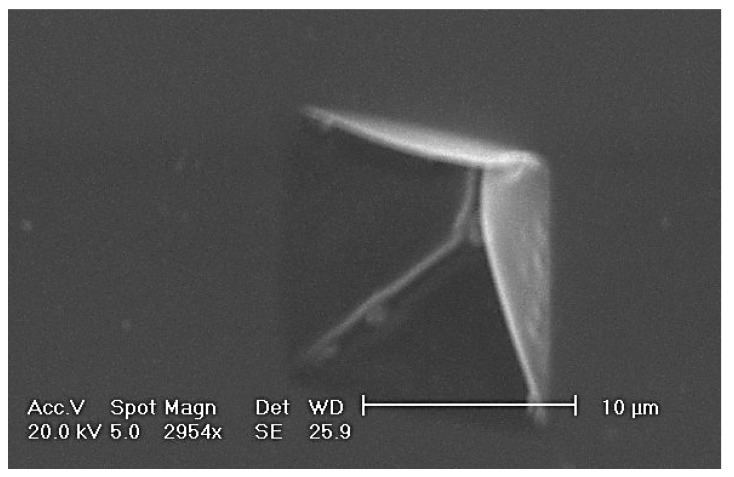
SEM image of a 10 μm × 10 μm freestanding pyramid sculpted on the surface of a metal-composite nanomembrane, thickness 8 nm.

An approach to surface sculpting of freestanding nanomembranes utilizing MEMS technologies was described in [69]. The fabrication procedure is schematically shown in Figure 21. The starting material is <100> Si single crystal. A two-step, double-sided etching process is used. Wet oxidation is first done and square windows are opened in the bottom side of SiO_2_. KOH etching was used to fabricate square silicon diaphragms 15–20 μm thick. Then another such process was performed from the top side and with much shorter etching procedure to fabricate arrays of micrometer-small pyramids in the diaphragm surface. In the next step, metal nanomembrane precursor (5–20 nm thin Cr film) is RF sputtered on the substrate in presence of oxygen or nitrogen, to form metal-composite. The pyramid shape on the top side is the consequence of the nature of anisotropic KOH etching. If isotropic etching is used, the shape of the holes on the top surface is spherical. After the Si diaphragm holding the precursor is fully dissolved, the deposited film is released either as a nanomembrane standing on the remaining Si rim or as a free-floating structure to be picked e.g., by a wire loop. A SEM image of a fabricated pyramid sculpted within the membrane is shown in Figure 22.

Alternatively, sculpting of nanomembrane can be done by placing the structure onto an elastic support and then mechanically stretching, corrugating, bending, *etc.* the support. Buckled (“wavy”) nanomembranes with a fishbone pattern were made of single crystalline silicon by Choi *et al.* [70]. They utilized elastomer support to adhere nanometer-thin single crystalline silicon membrane. In their experiment they started from silicon-on-insulator (SOI) wafers and thinned silicon to produce layers 3‑5 mm^2^ with a thickness between 55 nm and 320 nm. They cast and cured prepolymers of poly(dimethylsiloxane) (PDMS), made them hydrophilic by UV radiation and contacted them to the processed SOI until the whole Si nanomembrane was peeled off and transferred to the PDMS. Cooling the structure and thus releasing the thermally induced pre-strain spontaneously produced fishbone-patterned "waves" in silicon.

The most interesting fact is that since elastomer substrate is highly elastic, and so is silicon, it is possible to stretch the obtained structure along one (uniaxial) or both in-plane directions (biaxial) – the "ultracompliant" nanomembranes. If electronic devices (e.g., thin-film transistors) are produced in the silicon layer by conventional IC techniques, it is then possible to stretch them to desired dimension. Thus the described structures ensure a path to biaxially stretchable electronic devices.

A multilayer ultrathin 3D structure fabrication technique that can be used for corrugated nanomembrane formation prior its release is the autocloning [39]. This method was first proposed for the fabrication for ultrathin, 3D-formed photonic crystals.

## 10. Conclusions

In this paper, we have considered the methods and approaches to the modification of freestanding or free-floating nanomembranes in order to improve their applicability and tailor their functions. We proposed a classification of nanomembrane functionalization methods. The main approaches are lamination/stratification, surface activation and passivation, patterning (both additive and subtractive), pore formation, inclusion of nanofillers and 3D nano/microsculpting. Many of the described methods belong to MEMS and/or NEMS technologies. Obviously some of these nanocompositing and nanostructuring approaches will partly overlap (for instance, lamination and additive patterning, since such patterning may be interpreted as partial lamination; also surface activation, which may be also understood in some situations as lamination by a single monolayer of the modified material.

Any review covering a technology in full swing is necessarily partial and limited. One may argue that for the functionalization and generally modification of nanomembranes one may consider the whole ever expanding body of knowledge of nanotechnologies, naturally bearing in mind the peculiarities of the nanomembranes and their sensitivity to some of the fabrication methods.

A bright future could be expected for functionalized nanomembranes. Although we are literally at the beginning of the related investigations, the already existing achievements look extremely promising, and the rate of the appearance of new knowledge shows that we are on the increasing leg of an exponential curve. 

Practically it may be said that the applicability in this case equals functionalization. An example of what can be achieved by it is surely seen in living cells, where the existing processes sometimes appear to be on the verge of miraculous. The very fact that they exist means that at least in principle they could be replicated artificially. This is one of the tasks of the biomimetic branch of the nanotechnologies. A wider material and process toolbox which we have available should be theoretically able to do not only this, but to furnish improved and extended versions – or even completely new ones.

Similar to lipid bilayer nanomembranes, which are the basic building block of overall life while their functionalization is essential for their function and literally defines living organisms, one could envision an artificial nanomembrane as one of the basic building blocks of the MEMS of NEMS. Of course, it remains yet to be seen what can be done and what cannot, but the posed goal is sufficiently wide and important that this surely should be and will be done.
